# Epidemiology of Urological Cancers in Brazil: Trends in Mortality Rates Over More Than Two Decades

**DOI:** 10.1007/s44197-022-00042-8

**Published:** 2022-05-31

**Authors:** Thiago Camelo Mourão, Maria Paula Curado, Renato Almeida Rosa de Oliveira, Thiago Borges Marques Santana, Ricardo de Lima Favaretto, Gustavo Cardoso Guimarães

**Affiliations:** 1 Department of Uro-Oncology, BP-A Beneficência Portuguesa de São Paulo, São Paulo, Brazil; 2 Graduate School, Fundação Antônio Prudente, ACCamargo Cancer Center, R. Prof. Antônio Prudente, 211, São Paulo, 01509-010 Brazil; 3 Department of Statistics and Epidemiology, International Research Center, ACCamargo Cancer Center, São Paulo, Brazil; 4 Division of Urology, ACCamargo Cancer Center, São Paulo, Brazil; 5 Surgical Oncology Department, BP-A Beneficência Portuguesa de São Paulo, São Paulo, Brazil

**Keywords:** Urological cancers, Epidemiology, Mortality rates, Average annual percentage change

## Abstract

**Background:**

Considering the socioeconomic disparities and inequalities observed in the healthcare resources among the Brazilian regions, we aimed to analyze the mortality trends of urological cancers in Brazil to identify areas with differential risks.

**Methods:**

Deaths related to prostate (PCa), bladder (BCa), kidney (KC), penile (PeC), and testis (TCa) cancers from 1996 to 2019 were retrieved from the Mortality Information System database (Brazil). Geographic and temporal patterns were analyzed using age-standardized mortality rates (ASMRs). A joinpoint regression model was used to identify changes in the trends and calculate the average annual percentage change (AAPC) for each region.

**Results:**

In Brazil, the ASMRs (per 100,000 persons/year) were 11.76 for PCa; 1.37, BCa; 1.13, KC; 0.33, and PeC; 0.26, TCa over the period. Increasing mortality trends were registered for BCa (AAPC = 0.45 in men; 0.57 in women), KC (AAPC = 2.03 in men), PeC (AAPC = 1.01), and TCa (AAPC = 2.06). The PCa mortality presented a significant reduction after 2006. The Northeast and North regions showed the highest increases in the PCa mortality. The South registered the highest ASMRs for BCa and KC, but the highest increasing trends occurred in the men from the Northeast. The North presented the highest ASMR for PeC, while the South registered the highest ASMR for TCa.

**Conclusion:**

Differences among regions may be partly explained by disparities in the healthcare systems. Over the study period, the North and Northeast regions presented more discrepant mortality rates. Efforts should be made to ensure access to the healthcare resources for people at risk, particularly in these regions.

**Supplementary Information:**

The online version contains supplementary material available at 10.1007/s44197-022-00042-8.

## Introduction

The global aging population and the current population growth might partially explain the worldwide burdens of cancer incidence and mortality. In Brazil, cancer is the second major cause of death among those aged ≥ 70 years [1]. There are socioeconomic differences between Brazilian regions and cities, and these discrepancies influence the availability of diagnostic methods and treatments, determine migratory phenomena, and could possibly be related to differences in oncological outcomes [2].

According to the 2021 GLOBOCAN estimates, prostate cancer (PCa) represents 14.1% of malignant neoplasms in men, and 6.8% of the deaths caused by cancer (5th most common cause) [1]. Bladder cancer (BCa) deaths accounted for 2.1% of cancer-related deaths in both the sexes, with 440,864 new cases in men and 132,414, in women. Kidney cancer (KC) is also more common in men than in women, with 271,249 and 160,039 new cases, respectively. Testis cancer (TCa) and penile cancer (PeC) are less common, with new cases estimated at 0.4% and 0.2%, respectively. Each represents approximately 0.1% of cancer-related deaths [1].

Only a few published epidemiological studies have focused on urological malignancies and their distributions across the Brazilian regions. Understanding the epidemiology of these cancers could aid to elucidate potential regional risk factors and to suggest preventive strategies. Therefore, this study aimed to analyze the geographic distribution and time trends of urological cancers mortality in Brazil to identify areas with differential risks and outcomes for these neoplasms.

## Materials and Methods

### Data Source

This study analyzed the number of new cancer deaths retrieved from the Mortality Information System database (https://datasus.saude.gov.br) of the Brazilian Ministry of Health (SIM/MS) for the following urological malignancies and their respective ICD-10 codes: prostate (C61), bladder (C67), kidney (C64), testis (C62), and penile cancer (C60). Deaths registered between 1996 and 2019 were included in the analysis [3].

This is a free access database considered the main source of health information in Brazil. It provides tabulation tools that enable the researchers to select and organize the data according to the objectives of the study.

Data were obtained for the following variables: year of death, place of residence, age group, sex (if applied), and underlying cause of death according to the previously mentioned ICD-10 coding. All individuals included in the analysis were classified into the following age groups: 0–4, 5–9, 10–14, 15–19, 20–29, 30–39, 40–49, 50–59, 60–69, 70–79, and ≥ 80 years.

Population data were obtained from the webpage provided by the Brazilian Institute of Geography and Statistics for the census conducted in 2000 and 2010. For the intercensus years, estimates and projections published by the same data source were used [4]. Death cases with missing sex or age information were excluded from the analysis.

### Study Design and Geographical Areas

Geographic and temporal patterns were examined using age-standardized mortality rates (ASMRs), and they were expressed as per 100,000 persons per year. Adjustment by age was performed by the direct method, using the world standard population created by Segi in 1960 and modified by Doll in 1966 [5] as a reference for Brazil and the five geographic regions (North, Northeast, Central–West, Southeast, and South).

The Brazilian states and the Federal District are grouped into these five mentioned geographic regions. The population is highly concentrated in the Southeast region, with more than 40% of the Brazilian inhabitants living there, followed by the Northeast, with almost 30% of the population. The two most extensive regions are the Central-West and the North regions, which together represent 64% of the territory, but have a total of only 16% of the population [4].

Data on cancer deaths among men and women were extracted separately for bladder and kidney tumors. For BCa and KC mortality rates, an age-standardized rate ratio was calculated by dividing the ASMR for men by that for women in both neoplasias.

### Statistical Analysis

A joinpoint regression model was applied and permutation tests were performed to identify changes in the trends using the Joinpoint Regression Program for Windows, version 4.8.0.1 (National Cancer Institute, Bethesda, MD; available at https://surveillance.cancer.gov/joinpoint). The software enabled the user to test whether an apparent change in the trend is statistically significant. It fit the selected trend data into the simplest joinpoint model that the data allowed (maximum of four joinpoints) and calculated the average annual percentage change (AAPC) value for each region [6].

AAPC is a weighted average of the angular coefficients of the regression line with weights equal to the length of each segment over the interval. AAPC values were tested for equality to zero using the corresponding standard errors, and the values were considered statistically significant if *p* ≤ 0.05 [6]. Additionally, the maps were displayed using the software TabWin, version 4.15, provided by DATASUS, Brazil.

## Results

### Overall Mortality Rates of the Urological Cancers, 1996–2019

In Brazil, from 1996 to 2019, the SIM/MS database registered 270,117 deaths due to PCa; 70,690, BCa (48,993 in men, 21,689 in women); 54,021, KC (33,291 in men and 20,722 in women); 7371, PeC; and 6527, TCa.

For the entire period, the calculated ASMR in Brazil was 11.76 deaths per 100,000 men for PCa; 1.37, BCa; 1.13, KC; 0.33. PeC; and 0.26, for TCa. The age-specific mortality rates calculated for the periods 1996–2004 and 2005–2019 in Brazil are presented in Table 1. Maps showing the mean ASMRs in these two periods are also shown in Online Resources 1 and 2.

**Table 1:** Age-specific mortality rates of urological cancers in Brazil by age groups in the periods 1996–2004 and 2005–2019

	Age-specific mortality rate (1996–2004)^a^	Age-specific mortality rate (2005–2019)^b^
Prostate cancer		
< 50 years	0.09	0.12
50–59 years	6.52	6.60
60–69 years	40.90	43.87
70–79 years	151.05	170.10
80+ years	366.02	503.34
Bladder cancer (Male)		
< 50 years	0.09	0.10
50–59 years	2.47	2.42
60–69 years	9.25	9.89
70–79 years	25.33	28.87
80+ years	51.49	70.00
Bladder cancer (Female)		
< 50 years	0.06	0.06
50–59 years	1.09	1.22
60–69 years	2.88	3.43
70–79 years	7.38	8.67
80+ years	17.14	21.85
Kidney cancer (Male)		
< 50 years	0.24	0.29
50–59 years	3.06	3.91
60–69 years	6.16	9.32
70–79 years	10.12	14.92
80+ years	11.64	20.87
Kidney cancer (Female)		
< 50 years	0.18	0.20
50–59 years	1.50	1.77
60–69 years	3.18	4.01
70–79 years	5.71	6.97
80+ years	7.41	11.55
Penile cancer		
< 50 years	0.08	0.10
50–59 years	0.69	0.86
60–69 years	1.10	1.52
70–79 years	1.91	2.48
80+ years	4.16	5.40
Testis cancer		
< 30 years	0.16	0.26
30–39 years	0.40	0.58
40–49 years	0.29	0.31
50–59 years	0.18	0.25
60+ years	0.40	0.67

^a^Rates in 100,000 persons per year (based on census population 2000)

^b^Rates in 100,000 persons per year (based on census population 2010)

### Variations by Sex

When stratified by sex, the ASMR was higher in men than that in women for both BCa (2.18 deaths per 100,000 men and 0.74 deaths per 100,000 women) and KC (1.54 deaths per 100,000 men and 0.79 deaths per 100,000 women).

The standardized rate ratios (SRR) calculated between the sexes were represented by an SRR of 2.94 for BCa and 1.94 for KC, showing a predominance in men in both the cancers.

A detailed description of the ASMRs in each year of the period, with their respective standard errors (SE), is shown in Online Resources 3 and 4 for men and women, respectively.

### Regional Trends of Urological Cancers Mortality

**Fig. 1:**
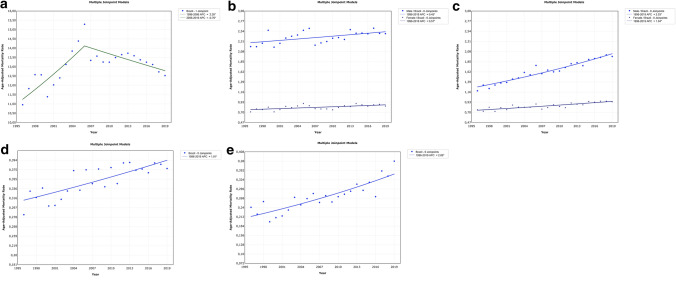
Trends in the age-standardized mortality rates for prostate (**a**), bladder (**b**), kidney (**c**), penile (**d**), and testicular (**e**) cancers in Brazil. *APC* annual percentage change

Increasing mortality trends were identified for PCa from 1996 to 2006, characterized by an annual percentage change (APC) of 2.3. This was followed by a slight but significant reduction from 2006 to 2019 (APC = − 0.76) (Fig. 1a). The peak ASMR for PCa in Brazil occurred in 2006 (15.29 deaths per 100,000 men).

**Fig. 2:**
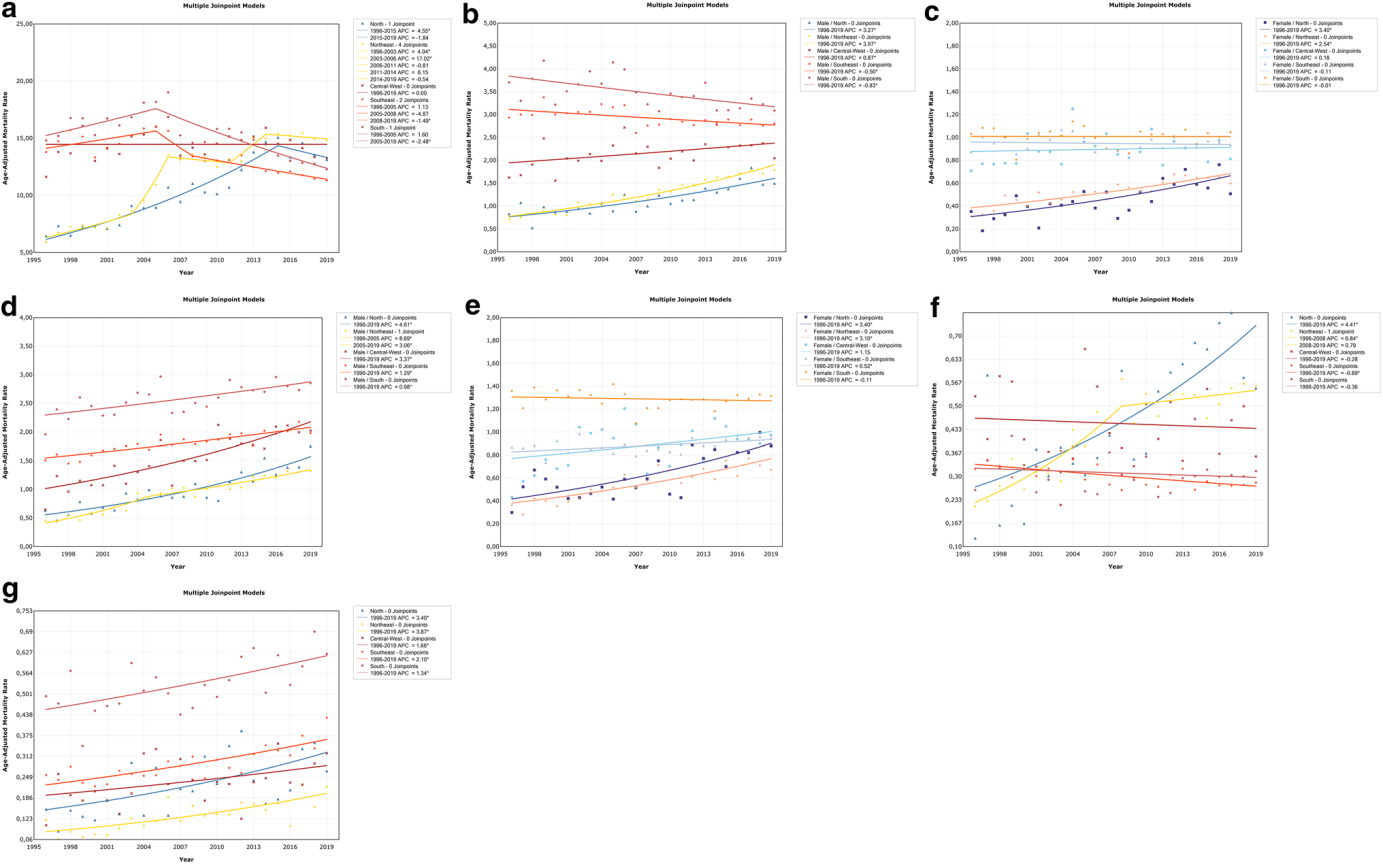
Trends in the age-standardized mortality rates for urological cancers in the Brazilian regions. **a** prostate cancer; **b** bladder cancer in men; **c** bladder cancer in women; **d** kidney cancer in men; **e** kidney cancer in women; **f** penile cancer; **g** testicular cancer. *APC* annual percentage change

In the Brazilian regions, the Northeast region presented the highest increase (Table 2), particularly in the period between 2003 and 2006 (APC = 17.02), followed by the North region. The Southern region showed a decreasing trend, predominantly after 2005. The Central–West region showed a similar ASMR during the entire period, and the Southeast region showed a significant reduction only after 2008 (APC = − 1.49), but this decreasing trend was not significant considering the full period (Fig. 2a and Table 2).

**Table 2:** Average annual percentage change of the age-standardized mortality rates of urological cancers among Brazilian regions between 1996 and 2019

	AAPC (1996–2019)	Lower 95% CI	Upper 95% CI	*p* value*
Prostate cancer				
Brazil	0.6*	1.0	2.5	**0.013**
North	3.4*	2.4	4.4	** < 0.001**
Northeast	3.8*	1.8	5.9	** < 0.001**
Central-West	0.0	− 0.5	0.5	0.996
Southeast	− 0.9	− 2.4	0.6	0.236
South	− 0.9*	− 1.7	− 0.1	**0.019**
Bladder cancer (Male)				
Brazil	0.5*	0.2	0.7	**0.004**
North	3.3*	2.4	4.2	** < 0.001**
Northeast	4.0*	3.5	4.4	** < 0.001**
Central-West	0.9*	0.1	1.7	**0.031**
Southeast	− 0.5*	− 0.8	− 0.2	**0.002**
South	− 0.8*	− 1.3	− 0.4	**0.001**
Bladder cancer (Female)				
Brazil	0.6*	0.3	0.9	**0.001**
North	3.4*	1.9	4.9	** < 0.001**
Northeast	2.5*	1.9	3.2	** < 0.001**
Central-West	0.2	− 0.7	1.1	0.682
Southeast	− 0.1	− 0.5	0.3	0.576
South	0.0	− 0.5	0.5	0.981
Kidney cancer (Male)				
Brazil	2.0*	1.8	2.3	** < 0.001**
North	4.6*	3.7	5.5	** < 0.001**
Northeast	5.2*	4.0	6.5	** < 0.001**
Central-West	3.4*	2.5	4.3	** < 0.001**
Southeast	1.3*	1.0	1.6	** < 0.001**
South	1.0*	0.6	1.4	** < 0.001**
Kidney cancer (Female)				
Brazil	1.0*	0.8	1.3	** < 0.001**
North	3.4*	2.1	4.7	** < 0.001**
Northeast	3.1*	2.3	3.9	** < 0.001**
Central-West	1.1	− 0.1	2.4	0.063
Southeast	0.5*	0.1	0.9	**0.011**
South	− 0.1	− 0.5	0.2	0.516
Penile cancer				
Brazil	1.0*	0.7	1.3	** < 0.001**
North	4.4*	2.6	6.3	** < 0.001**
Northeast	3.9*	2.6	5.2	** < 0.001**
Central-West	− 0.3	− 1.7	1.1	0.682
Southeast	− 0.9*	− 1.4	− 0.4	**0.001**
South	− 0.4	− 1.2	0.5	0.363
Testis cancer				
Brazil	2.1*	1.6	2.6	** < 0.001**
North	3.4*	1.3	5.6	**0.003**
Northeast	3.9*	2.4	5.4	** < 0.001**
Central-West	1.7*	0.0	3.3	**0.047**
Southeast	2.1*	1.5	2.7	** < 0.001**
South	1.3*	0.6	2.1	**0.001**

*AAPC* average annual percent change, *CI* confidence interval

*Bold values indicate that the average annual percentage change (AAPC) is significantly different from zero (*p* ≤ 0.05)

Regarding the mortality trends of BCa, the peak ASMR was 2.61 deaths per 100,000 men, with an increasing trend in the period of the study (AAPC = 0.45) and 0.9 per 100,000 women (AAPC = 0.57) (Fig. 1b). The South region had the highest ASMR, considering both men and women (Fig. 2b, c); but among men, the South region showed a significant reduction over the period (AAPC = − 0.8). The North and Northeast regions had increasing mortality trends in both the sexes, particularly in men in the Northeast, in comparison to the national data (Table 3).

**Table 3:** Correlations of the average annual percentage change of the age-standardized mortality rates of urological cancers among Brazil and the Brazilian regions between 1996–2019

	AAPC (1996–2019)	Lower 95% CI	Upper 95% CI	*p* value*
Prostate cancer				
Brazil vs North	− 2.9*	− 4.0	− 1.7	** < 0.001**
Brazil vs Northeast	− 3.3*	− 5.3	− 1.2	**0.002**
Brazil vs Central-West	0.6	− 0.1	1.2	0.088
Brazil vs Southeast	1.5	− 0.1	3.1	0.066
Brazil vs South	1.5*	0.6	2.3	**0.001**
Bladder cancer (Male)				
Brazil vs North	− 2.8*	− 3.7	− 1.9	** < 0.001**
Brazil vs Northeast	− 3.5*	− 4.0	− 3.0	** < 0.001**
Brazil vs Central-West	− 0.4	− 1.2	0.4	0.299
Brazil vs Southeast	1.0*	0.6	1.3	** < 0.001**
Brazil vs South	1.3*	0.8	1.8	** < 0.001**
Bladder cancer (Female)				
Brazil vs North	− 2.8*	− 4.3	− 1.4	** < 0.001**
Brazil vs Northeast	− 2.0*	− 2.6	− 1.3	** < 0.001**
Brazil vs Central-West	0.4	− 0.5	1.3	0.375
Brazil vs Southeast	0.7*	0.2	1.2	**0.005**
Brazil vs South	0.6*	0.0	1.1	**0.035**
Kidney cancer (Male)				
Brazil vs North	− 2.6*	− 3.5	− 1.7	** < 0.001**
Brazil vs Northeast	− 3.2*	− 4.5	− 1.9	** < 0.001**
Brazil vs Central-West	− 1.3*	− 2.2	− 0.4	**0.003**
Brazil vs Southeast	0.7*	0.4	1.1	** < 0.001**
Brazil vs South	1.1*	0.6	1.5	** < 0.001**
Kidney cancer (Female)				
Brazil vs North	− 2.4*	− 3.6	− 1.1	** < 0.001**
Brazil vs Northeast	− 2.1*	− 2.9	− 1.2	** < 0.001**
Brazil vs Central-West	− 0.1	− 1.3	1.1	0.854
Brazil vs Southeast	0.5*	0.1	1.0	**0.025**
Brazil vs South	1.1*	0.7	1.6	** < 0.001**
Penile cancer				
Brazil vs North	− 3.4*	− 5.2	− 1.6	** < 0.001**
Brazil vs Northeast	− 2.9*	− 4.2	− 1.6	** < 0.001**
Brazil vs Central-West	1.3	− 0.1	2.6	0.061
Brazil vs Southeast	1.9*	1.4	2.4	** < 0.001**
Brazil vs South	1.4*	0.6	2.2	**0.001**
Testis cancer				
Brazil vs North	− 1.3	− 3.4	0.7	0.208
Brazil vs Northeast	− 1.8*	− 3.3	− 0.3	**0.017**
Brazil vs Central-West	0.4	− 1.2	2.0	0.633
Brazil vs Southeast	0.0	− 0.8	0.7	0.931
Brazil vs South	0.7	− 0.1	1.6	0.089

*AAPC* average annual percent change, *CI* confidence interval

*Bold values indicate that the average annual percentage change (AAPC) is significantly different from zero (*p* ≤ 0.05)

The ASMR for KC showed upward mortality trends in both sexes, particularly in men (AAPC = 2.03) (Fig. 1c). This trend was higher among men in the Northeast region, followed by the North and Central–West regions (Fig. 2d). In women, the growing trend was highest and significant in the north, followed by the Northeast and Southeast. Despite this, the South region also registered the highest ASMRs in the period for both the sexes. Considering all the groups, only women in the South showed a decreasing mortality trend, but this was not statistically significant (AAPC = − 0.11) (Fig. 2e and Table 2).

There were fewer deaths due to PeC and TCa than those due to other urological cancers. Both registered increasing mortality trends (AAPC = 1.01 and 2.06, respectively) (Fig. 1d, e). As expected, the North and Northeast regions had the highest ASMRs and AAPCs for PeC. At the end of this period, the North region was the most impacted, particularly after the stagnation of the ASMR in the Northeast region since 2008 (Fig. 2f). A significant decreasing trend was identified only in the Southeast region. As compared to the national trend, the Southeast region presented the most negative difference (Table 3).

Regarding TCa, all the regions showed significant upward trends, but the ASMR was the highest in the South (Fig. 2g). As compared to the Brazilian data, a significant difference in AAPC was observed only in the Northeast (Table 3).

## Discussion

This study provides an overview of mortality patterns and trends of the main urological malignancies in Brazil and its regions. To the best of our knowledge, it is the most comprehensive analysis of mortality data in urological malignancies. In the last 2 decades, except for PCa, all urological cancers showed upward trends in the mortality rates. A particular observed point was that the North and Northeast were the regions that more contributed to these increases. These regions still demonstrate the highest rates of poverty and the areas with the lowest Human Development Indices in the country [7].

The rise in mortality rates for PCa in Brazil at the end of the 1990s and the beginning of the 2000s was also noted in previous population studies [8–12]. However, it has occurred late as compared to developed countries such as the United States, Canada, the United Kingdom, Germany, and France [9, 13]. In our study, a reduction in PCa mortality occurred only from 2006 onwards (APC 2006–2019 = − 0.8; *p* = 0.003). Several reasons might explain our results, likely reflecting increased awareness and advancements in healthcare structure, enabling a late but gradually broader adoption of prostate-specific antigen testing and biopsies [14, 15]. Contemporary analyses have suggested that decreasing trends will continue to occur in Brazil [11, 16].

A study conducted by Jerez-Roig et al*.* (2014) observed a stable plateau in PCa mortality from 2006 to 2010, in contrast to a significant increase in the North region. The authors performed a projection from 2011–2025, reporting that the increasing ASMRs in the North and Northeast regions were mainly attributed to population changes [10]. In contrast, we noted non-significant changes in the Northeast region since 2006 and in the North region since 2015. Nevertheless, the AAPC across the whole period showed increasing trends in these regions, and structural changes certainly persisted. Due to fewer oncological services in these regions, prostate screening tends to be less prevalent among men with lower schooling levels and low income, indicating a potential increase in the absolute number of deaths [10].

Regarding the role of ethnicity in the prognosis of PCa, men of African and Caribbean descent had the highest incidence and mortality rates [1]. The South region presents the highest proportion of Caucasians in Brazil (approximately 80%). Due to the complexity of the miscegenation phenomenon in Brazil, it is unclear if this could explain the most significant decrease observed in this region in comparison to the national data.

Bladder cancer is the seventh most common cancer in Brazilian men and is ranked 16th among women. Similarly, mortality is also higher among men than in women (12th and 16th, respectively) [8]. Geographic and temporal patterns of BCa mortality show trends similar to those of tobacco consumption. Antoni et al*.* (2017) reported that BCa incidence and mortality were higher in countries with very high or high human development indices, but a decreasing trend in mortality has been observed, except in transition economies such as South American countries, where smoking prevalence started decreasing more recently [17, 18].

In this way, it is possible that decreases in BCa rates will not be observed in the North and Northeast regions for a few decades. Concurrently, the prevalence of smoking in the overall Brazilian population has declined by more than 2% per year in recent years [19], but a growing prevalence has been observed among women [20, 21]. Our analysis showed that the mortality trend decreased in men from the Southeast and South regions. On the contrary, this variation was not observed in women in these regions.

The Northeast and North regions showed a worsening of the BCa burden in Brazil in both the sexes. Successful public policies for tobacco consumption need to be implemented to bring about further declines in these rates, in addition to improving access to the health facilities offered to these people [19, 20, 22].

Growing mortality trends for KC were also reported by Sierra et al. in Brazil from 1997 to 2006 in both the sexes (AAPC = 2.7, men; 1.0, women) [8]. The worldwide incidence and mortality rates varied and were higher in the developed areas. Known risk factors include smoking, obesity, and hypertension [23]. Except Southern and Central-Western women, all the other groups showed increasing mortality trends, particularly people in the North and Northeast. However, there is a predominance of deaths due to KC in people originating from the South during the entire period. The role of smoking prevalence or genotypes in the Southern region could not be assessed in this study.

It is well documented that PeC is more prevalent in developing countries, and it is a highly aggressive urogenital tumor. The most important risk factors among men with phimosis or excess prepuce are low socioeconomic level and poor hygiene, in addition to a history of sexually transmitted diseases (STDs), and a high prevalence of human papillomavirus (HPV) infection in penile cancer, reaching approximately 50% [24–26]. In the current study, only the Southeast region demonstrated a significant reduction in the ASMR. A previous epidemiologic study predicted an increasing ASMR over all the age groups in Brazil up to 2025, particularly in men aged > 50 years [27]. Considering the period between 1996 and 2019, our analysis evidenced an ASMR of 0.43 and 0.40 per 100,000 men in the North and Northeast regions, respectively.

A delay in diagnosis, associated with the limited access to healthcare, results in a worse prognosis. A recently published Brazilian consensus reported that circumcision, smoking reduction, and educational campaigns regarding proper hygiene habits, STD prevention, and HPV vaccination can decrease PeC rates [25]. Efforts to promote male health through a national policy for comprehensive male healthcare must be stimulated [26]. Currently, the quadrivalent HPV vaccine is available through the public health system in Brazil for girls aged 9–14 years, boys aged 11–14 years, immunosuppressed men aged up to 26 years, and immunosuppressed women aged up to 45 years.

Finally, TCa is considered rare, but it is one of the most frequently diagnosed cancers in young men aged < 40 years. TCa can be aggressive, but it usually presents a high chance of cure when diagnosed in the early stages [28]. The ASMR in the period of 1996–2019 (0.26 per 100,000 men) was similar to that reported in previous studies [29]. This malignancy is more common in developed areas. The highest ASMR occurred in the South. However, the highest AAPC was observed in the Northeastern region.

A recent publication that predicted data from 2016 to 2030 described stability in ASMR only for the Southeast region, in addition to a slight reduction in rates for the Central–West region [29]. In contrast, our study showed a constant growing trend in all regions from 1996–2019. The main reasons for regional differences are related to socioeconomic features and the availability of healthcare services. Ethnicity is a risk factor that can be assessed. The high proportion of Caucasians in the South can partly explain the disparity in mortality rates. Furthermore, improving the efficacy of protocols and educating men in favor of self-examination could aid in the early diagnosis.

The interpretation of our data demands some caution, as the absolute numbers are based on the availability and accuracy of each regional data. Previous studies have reported that mortality data may be less accurate among elderly groups due to the challenges in identifying the cause of death in this group of patients with multiple comorbidities [30]. Inaccuracies among elderly patients, particularly those with PCa, KC, and BCa, could affect the final measure. Individual factors were not available for a comprehensive evaluation of this type of study. In addition, delays in epidemiological reports, coding errors, and limited resources could limit the reliability of the number of deaths [31].

There were concerns regarding the reliability of the Brazilian registry of deaths, particularly in the North and Northeast regions. Nevertheless, improvements and standardization of data collection and registration have been implemented in the last two decades [10, 32].

In conclusion, this study provides an overview of mortality patterns and trends of the main urological malignancies in Brazil and its regions using public data retrieved from SIM/MS over the last two decades. This public information system can serve as a tool for creating public healthcare policies. Differences observed in ASMR and mortality trends among regions may be partly explained by disparities in healthcare systems, access to treatment, and diagnosis in reference cancer centers. Additionally, the national aging phenomenon must continue to influence the rise in mortality rates in Brazil.

Healthcare providers and policy makers should ﻿ensure health policies to guarantee access for elderly people, particularly in the North and Northeast regions, where increasing and discrepant mortality rates were observed across the study period.

## Supplementary Information

Below is the link to the electronic supplementary material.

Online Resource 3: Distribution of annual age-standardized mortality rates for urological cancers in Brazilian men from 1996 to 2019 (DOCX 32 KB)

Online Resource 4: Distribution of the annual age-standardized mortality rates for bladder and kidney cancers in Brazilian women from 1996 to 2019 (DOCX 19 KB)

## Data Availability

Raw data are available for conferences upon request. All data were retrieved from the public Brazilian Mortality Information System available at https://datasus.saude.gov.br.

## Abbreviations

AAPC: Average annual percentage change
APC: Annual percentage change
ASMR: Age-standardized mortality rate
BCa: Bladder cancer
GLOBOCAN: Global Cancer Observatory
KC: Kidney cancer
PCa: Prostate cancer
PeC: Penile cancer
SRR: Standardized rate ratio
STD: Sexually transmitted disease
TCa: Testicular cancer

## References

[1] Sung H, Ferlay J, Siegel RL, Laversanne M, Soerjomataram I, Jemal A (2021). Global cancer Statistics 2020: GLOBOCAN estimates of incidence and mortality worldwide for 36 cancers in 185 countries. CA Cancer J Clin.

[2] Cubero DIG, Sette CVM, Piscopo BCP, Monteiro CRA, Schoueri JHM, Tavares HDA (2018). Epidemiological profile of Brazilian oncological patients seen by a reference oncology center of the public health system and who migrate in search of adequate health care. Rev Assoc Med Bras.

[3] DATASUS, Department of Informatics, Ministry of Health, Brazil. Information in Health: Mortality Information System. http://tabnet.datasus.gov.br/cgi/deftohtm.exe?sim/cnv/obt10uf.def. Accessed 10 Feb 2021.

[4] DATASUS, Department of Informatics, Ministry of Health, Brazil. Information in Health: Demographic and Socioeconomic. http://www2.datasus.gov.br/DATASUS/index.php?area=0206&id=6942. Accessed 10 Feb 2021.

[5] Doll R, Cook P (1967). Summarizing indices for comparison of cancer incidence data. Int J Cancer.

[6] Kim HJ, Fay MP, Feuer EJ, Midthune DN (2000). Permutation tests for joinpoint regression with applications to cancer rates. Stat Med.

[7] Soares S, de Souza L, Silva WJ, Silveira FG. Poverty profile: the rural North and Northeast of Brazil. In: IPC-IG Working Paper No. 138. International Policy Centre for Inclusive Growth, Brasília. 2016. https://ipcig.org/sites/default/files/pub/en/PRB50_Poverty_profile_the_rural_North_Northeast_regions_of_Brazil.pdf. Accessed 15 Feb 2022

[8] Sierra MS, Soerjomataram I, Antoni S, Laversanne M, Piñeros M, de Vries E (2016). Cancer patterns and trends in Central and South America. Cancer Epidemiol.

[9] Fonseca LAM, Eluf-Neto J, Wunsch FV (2010). Cancer mortality trends in Brazilian state capitals, 1980–2004. Rev Assoc Med Bras.

[10] Jerez-Roig J, Souza DLB, Medeiros PFM, Barbosa IR, Curado MP, Costa ICC (2014). Future burden of prostate cancer mortality in Brazil: a population-based study. Cad Saude Publica.

[11] Serpa Neto A, Tobias-Machado M, Wroclawski ML, Akerman M, Pompeo ACL, Giglio AD (2010). A descriptive study of prostate cancer mortality in the state of São Paulo, from 1980 to 2007. Einstein (São Paulo).

[12] Luizaga CTM, Ribeiro KB, Fonseca LAM, Eluf NJ (2020). Trends in prostate cancer mortality in the state of São Paulo, 2000 to 2015. Rev Saúde Publ.

[13] Collin SM, Martin RM, Metcalfe C, Gunnell D, Albertsen PC, Neal D (2008). Prostate-cancer mortality in the USA and UK in 1975–2004: an ecological study. Lancet Oncol.

[14] Bray F, Piñeros M (2016). Cancer patterns, trends and projections in Latin America and the Caribbean: a global context. Salud Publica Mex.

[15] Seraphin TP, Joko-Fru WY, Kamaté B, Chokunonga E, Wabinga H, Somdyala NIM (2021). Rising prostate cancer incidence in sub-Saharan Africa: a trend analysis of data from the African cancer registry network. Cancer Epidemiol Biomark Prev.

[16] Culp MB, Soerjomataram I, Efstathiou JA, Bray F, Jemal A (2020). Recent global patterns in prostate cancer incidence and mortality rates. Eur Urol.

[17] Antoni S, Ferlay J, Soerjomataram I, Znaor A, Jemal A, Bray F (2017). Bladder cancer incidence and mortality: a global overview and recent trends. Eur Urol.

[18] Global Burden of Disease Cancer Collaboration (2017). Global, regional, and national cancer incidence, mortality, years of life lost, years lived with disability, and disability-adjusted life-years for 32 cancer groups, 1990 to 2015: a systematic analysis for the global burden of disease study. JAMA Oncol.

[19] Monteiro CA, Cavalcante TM, Moura EC, Claro RM, Szwarcwald CL (2007). Population-based evidence of a strong decline in the prevalence of smokers in Brazil (1989–2003). Bull World Health Organ.

[20] GBD 2015 Tobacco Collaborators. Smoking prevalence and attributable disease burden in 195 countries and territories, 1990–2015: a systematic analysis from the Global Burden of Disease Study 2015. Lancet. 2017;389:1885–906. 10.1016/S0140-6736(17)30819-X 10.1016/S0140-6736(17)30819-X PMC5439023 28390697

[21] Islami F, Goding Sauer A, Miller KD, Siegel RL, Fedewa SA, Jacobs EJ (2018). Proportion and number of cancer cases and deaths attributable to potentially modifiable risk factors in the United States. CA Cancer J Clin.

[22] Fernandes GA, Algranti E, Conceição GMS, Wünsch Filho V, Toporcov TN (2019). Lung cancer mortality trends in a Brazilian city with a long history of asbestos consumption. Int J Environ Res Public Health.

[23] Capitanio U, Bensalah K, Bex A, Boorjian SA, Bray F, Coleman J (2019). Epidemiology of renal cell carcinoma. Eur Urol.

[24] Favorito LA, Nardi AC, Ronalsa M, Zequi SC, Sampaio FJB, Glina S (2008). Epidemiologic study on penile cancer in Brazil. Int Braz J Urol.

[25] Soares A, de Carvalho IT, da Fonseca AG, Alencar AM, Leite CHB, Bastos DA (2020). Penile cancer: a Brazilian consensus statement for low- and middle-income countries. J Cancer Res Clin Oncol.

[26] Korkes F, Rodrigues AFS, Baccaglini W, Cunha FTS, Slongo J, Spiess P, et al. Penile cancer trends and economic burden in the Brazilian public health system. Einstein (São Paulo). 2020;18:eAO5577; 10.31744/einstein_journal/2020AO5577 10.31744/einstein_journal/2020AO5577 PMC7607917 33174969

[27] de Souza DLB, Curado MP, Bernal MM, Jerez-Roig J, Boffetta P (2013). Mortality trends and prediction of HPV-related cancers in Brazil. Eur J Cancer Prev.

[28] Smith ZL, Werntz RP, Eggener SE (2018). Testicular cancer: epidemiology, diagnosis, and management. Med Clin N Am.

[29] Soares SCM, dos Santos KMR, de Morais Fernandes FCG, Barbosa IR, de Souza DLB (2019). Testicular Cancer mortality in Brazil: trends and predictions until 2030. BMC Urol.

[30] Pilleron S, Soerjomataram I, Soto-Perez-de-Celis E, Ferlay J, Vega E, Bray F (2019). Aging and the cancer burden in Latin America and the Caribbean: time to act. J Geriatr Oncol.

[31] Hashim D, Boffetta P, La Vecchia C, Rota M, Bertuccio P, Malvezzi M (2016). The global decrease in cancer mortality: trends and disparities. Ann Oncol.

[32] Chatenoud L, Bertuccio P, Bosetti C, Levi F, Curado MP, Malvezzi M (2010). Trends in cancer mortality in Brazil, 1980–2004. Eur J Cancer Prev.

