# Supplementary figures and images for: Epidemiology of Urological Cancers in Brazil: Trends in Mortality Rates Over More Than Two Decades
Source: J Epidemiol Glob Health. 2022 May 31;12(3):239–47. doi: 10.1007/s44197-022-00042-8 (PMC9470798; doi:10.1007/s44197-022-00042-8)

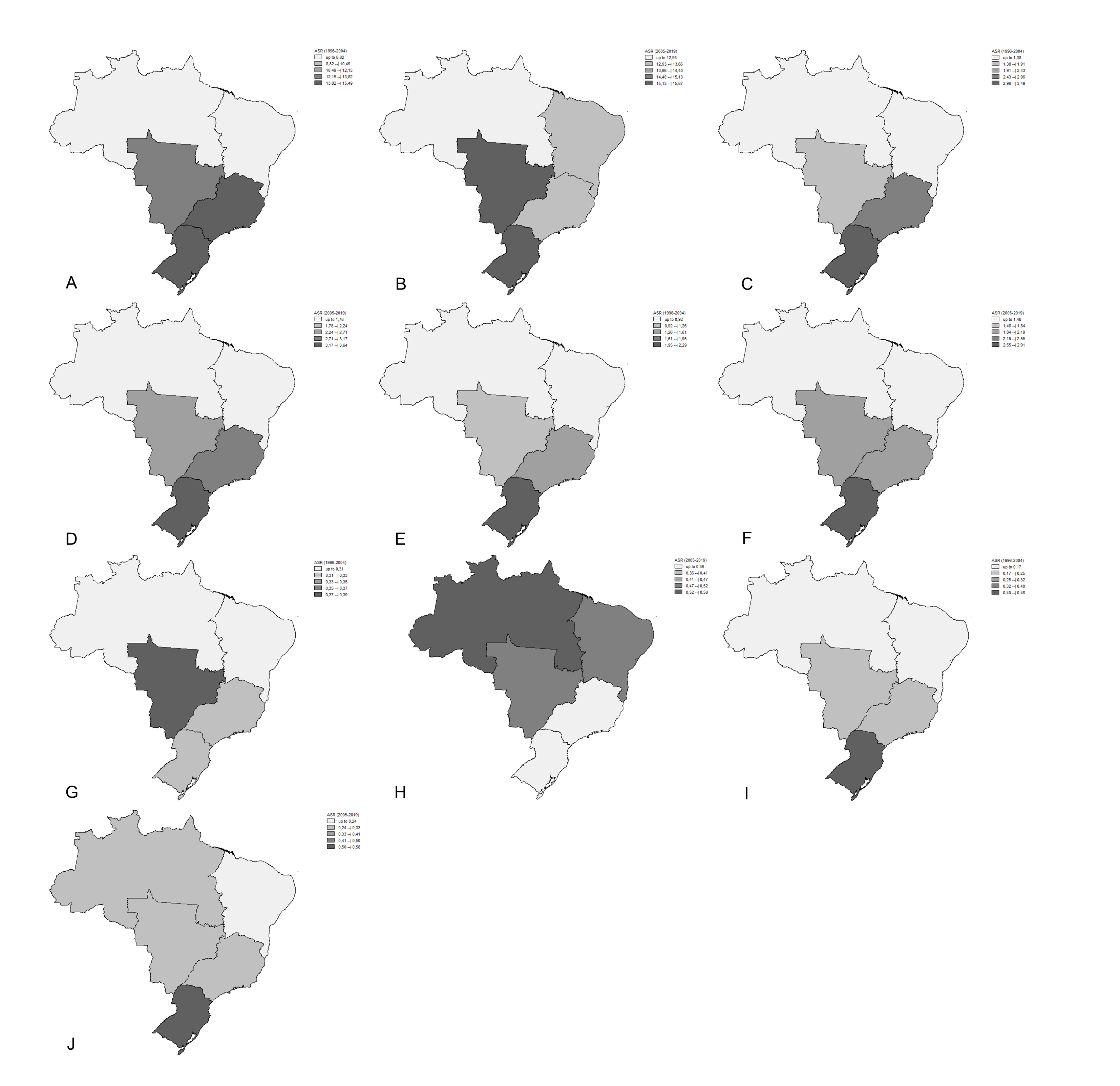

Supplement: Supplementary file 1 [file 44197_2022_42_MOESM1_ESM.tif]

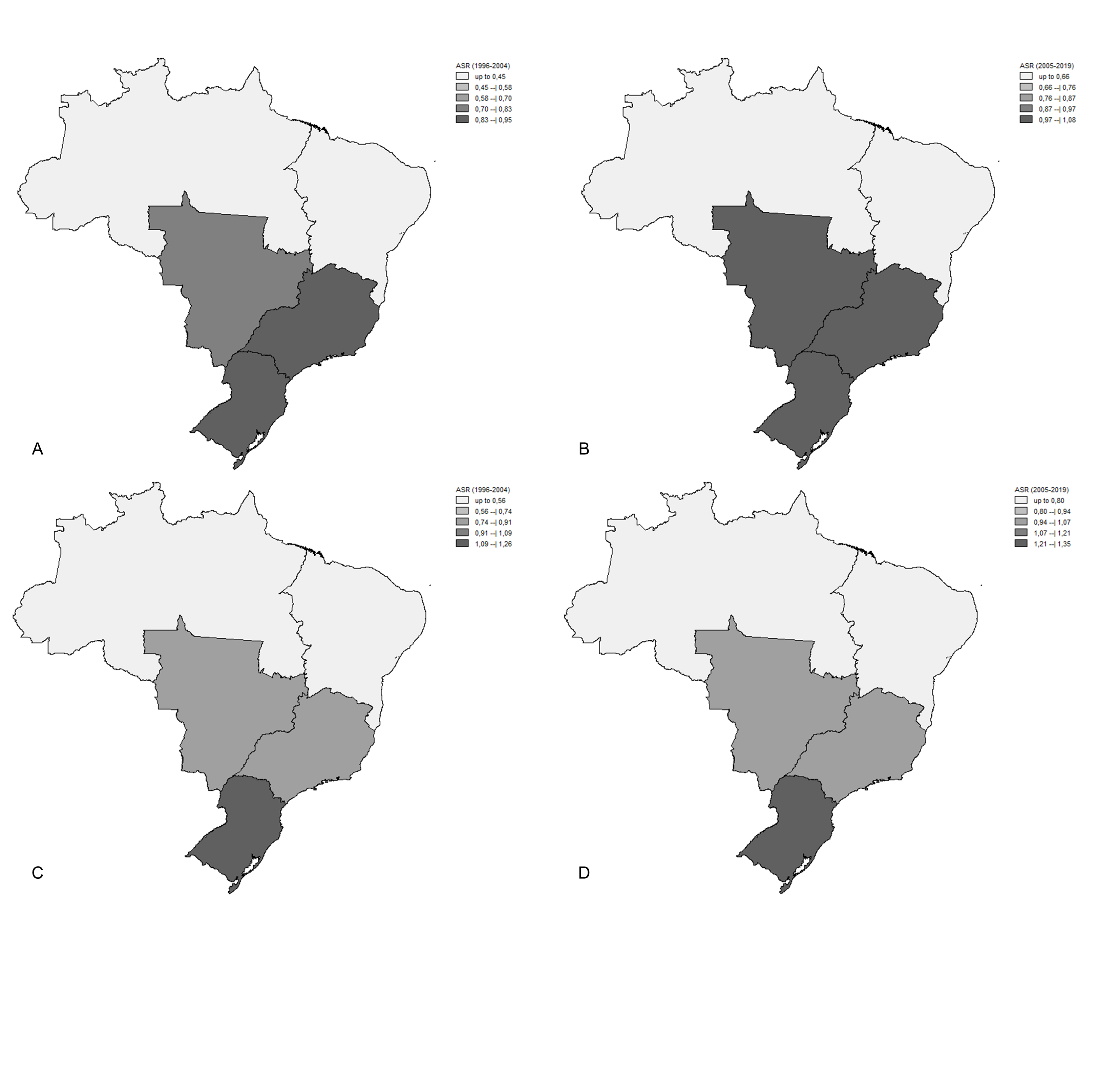

Supplement: Supplementary file 2 [file 44197_2022_42_MOESM2_ESM.tif]
