# Supplementary material for: Epidemiology of Urological Cancers in Brazil: Trends in Mortality Rates Over More Than Two Decades
Source: J Epidemiol Glob Health. 2022 May 31;12(3):239–47. doi: 10.1007/s44197-022-00042-8 (PMC9470798; doi:10.1007/s44197-022-00042-8)
Supplement: Supplementary file 3 — Online Resource 3: Distribution of annual age-standardized mortality rates for urological cancers in Brazilian men from 1996 to 2019 (DOCX 32 KB) [file 44197_2022_42_MOESM3_ESM.docx]

|  | **Region** | **1996** | **1997** | **1998** | **1999** | **2000** | **2001** | **2002** | **2003** | **2004** | **2005** | **2006** | **2007** | **2008** | **2009** | **2010** | **2011** | **2012** | **2013** | **2014** | **2015** | **2016** | **2017** | **2018** | **2019** |
| --- | --- | --- | --- | --- | --- | --- | --- | --- | --- | --- | --- | --- | --- | --- | --- | --- | --- | --- | --- | --- | --- | --- | --- | --- | --- |
| **Bladder cancer** |  |  |  |  |  |  |  |  |  |  |  |  |  |  |  |  |  |  |  |  |  |  |  |  |  |
| Male | **Brazil** | 2.19 (0.06) | 2.19 (0.06) | 2.27 (0.06) | 2.56 (0.07) | 2.18 (0.06) | 2.27 (0.06) | 2.39 (0.06) | 2.43 (0.06) | 2.46 (0.06) | 2.56 (0.06) | 2.61 (0.06) | 2.23 (0.05) | 2.28 (0.05) | 2.31 (0.05) | 2.38 (0.05) | 2.4 (0.05) | 2.36 (0.05) | 2.58 (0.05) | 2.5 (0.05) | 2.5 (0.05) | 2.48 (0.05) | 2.61 (0.05) | 2.49 (0.05) | 2.49 (0.04) |
|  | North | 0.83 (0.17) | 1.07 (0.19) | 0.52 (0.13) | 0.98 (0.19) | 0.87 (0.16) | 0.88 (0.16) | 0.95 (0.16) | 0.84 (0.15) | 1.06 (0.17) | 0.9 (0.15) | 1.25 (0.17) | 0.89 (0.14) | 1.0 (0.15) | 1.24 (0.16) | 1.06 (0.14) | 1.13 (0.14) | 1.14 (0.14) | 1.39 (0.16) | 1.3 (0.15) | 1.38 (0.15) | 1.61 (0.16) | 1.84 (0.17) | 1.47 (0.15) | 1.5 (0.15) |
|  | Northeast | 0.71 (0.07) | 0.76 (0.07) | 0.83 (0.07) | 0.89 (0.07) | 0.81 (0.07) | 0.8 (0.07) | 1.09 (0.08) | 1.05 (0.07) | 1.03 (0.07) | 1.25 (0.08) | 1.22 (0.08) | 1.12 (0.07) | 1.15 (0.07) | 1.36 (0.08) | 1.31 (0.07) | 1.47 (0.08) | 1.47 (0.08) | 1.58 (0.08) | 1.63 (0.08) | 1.64 (0.08) | 1.71 (0.08) | 1.79 (0.08) | 1.71 (0.08) | 1.79 (0.08) |
|  | Center-West | 1.62 (0.23) | 1.68 (0.24) | 1.91 (0.25) | 2.48 (0.28) | 1.56 (0.21) | 2.04 (0.23) | 1.99 (0.23) | 2.14 (0.23) | 1.99 (0.23) | 2.32 (0.24) | 2.72 (0.26) | 2.15 (0.21) | 2.3 (0.21) | 1.84 (0.18) | 2.04 (0.19) | 2.2 (0.19) | 2.0 (0.18) | 2.35 (0.19) | 2.78 (0.2) | 2.29 (0.18) | 2.32 (0.18) | 2.33 (0.17) | 2.37 (0.17) | 2.05 (0.16) |
|  | Southeast | 2.94 (0.11) | 3.3 (0.11) | 2.99 (0.11) | 3.38 (0.12) | 3.0 (0.1) | 3.03 (0.1) | 3.06 (0.1) | 3.06 (0.1) | 3.23 (0.1) | 3.16 (0.1) | 3.21 (0.1) | 2.6 (0.08) | 2.76 (0.08) | 2.78 (0.08) | 2.9 (0.08) | 2.84 (0.08) | 2.77 (0.08) | 2.88 (0.08) | 2.84 (0.08) | 2.89 (0.08) | 2.77 (0.07) | 2.89 (0.07) | 2.76 (0.07) | 2.8 (0.07) |
|  | South | 3.7 (0.21) | 3.3 (0.2) | 3.79 (0.21) | 4.18 (0.22) | 3.22 (0.18) | 3.51 (0.19) | 3.66 (0.19) | 3.95 (0.2) | 3.68 (0.19) | 4.14 (0.2) | 3.99 (0.19) | 3.49 (0.16) | 3.23 (0.15) | 3.07 (0.15) | 3.46 (0.16) | 3.38 (0.15) | 3.41 (0.15) | 3.7 (0.15) | 3.09 (0.14) | 3.1 (0.13) | 3.14 (0.13) | 3.35 (0.13) | 3.23 (0.13) | 3.09 (0.12) |
| **Kidney cancer** |  |  |  |  |  |  |  |  |  |  |  |  |  |  |  |  |  |  |  |  |  |  |  |  |  |
| Male | **Brazil** | 1.19 (0.03) | 1.32 (0.03) | 1.24 (0.03) | 1.32 (0.03) | 1.37 (0.03) | 1.38 (0.03) | 1.46 (0.03) | 1.48 (0.03) | 1.61 (0.03) | 1.55 (0.03) | 1.76 (0.03) | 1.58 (0.03) | 1.66 (0.03) | 1.62 (0.03) | 1.64 (0.03) | 1.72 (0.03) | 1.8 (0.03) | 1.83 (0.03) | 1.76 (0.03) | 1.9 (0.03) | 1.92 (0.02) | 1.95 (0.02) | 2.0 (0.02) | 1.97 (0.02) |
|  | North | 0.63 (0.11) | 0.46 (0.08) | 0.56 (0.1) | 0.79 (0.11) | 0.59 (0.12) | 0.69 (0.11) | 0.64 (0.07) | 0.94 (0.1) | 0.83 (0.1) | 0.99 (0.11) | 0.89 (0.11) | 0.86 (0.09) | 0.88 (0.11) | 1.1 (0.07) | 0.86 (0.08) | 0.81 (0.1) | 1.14 (0.09) | 1.31 (0.11) | 1.15 (0.1) | 1.55 (0.11) | 1.26 (0.1) | 1.39 (0.09) | 1.39 (0.1) | 1.75 (0.08) |
|  | Northeast | 0.46 (0.04) | 0.44 (0.04) | 0.54 (0.04) | 0.46 (0.05) | 0.59 (0.05) | 0.56 (0.04) | 0.73 (0.05) | 0.63 (0.04) | 0.93 (0.05) | 0.85 (0.05) | 0.94 (0.05) | 1.02 (0.05) | 1.03 (0.04) | 0.87 (0.05) | 1.01 (0.04) | 1.05 (0.04) | 1.01 (0.04) | 1.03 (0.04) | 1.22 (0.05) | 1.24 (0.05) | 1.21 (0.04) | 1.32 (0.04) | 1.3 (0.04) | 1.32 (0.04) |
|  | Center-West | 0.65 (0.15) | 1.23 (0.16) | 0.96 (0.15) | 1.15 (0.16) | 1.07 (0.14) | 1.07 (0.16) | 1.41 (0.15) | 1.1 (0.14) | 1.31 (0.13) | 1.41 (0.17) | 1.86 (0.15) | 1.07 (0.13) | 1.5 (0.13) | 1.5 (0.12) | 1.51 (0.11) | 2.12 (0.11) | 1.88 (0.13) | 1.8 (0.1) | 1.78 (0.11) | 1.71 (0.11) | 2.09 (0.1) | 2.11 (0.09) | 2.0 (0.1) | 2.02 (0.09) |
|  | Southeast | 1.51 (0.05) | 1.61 (0.06) | 1.45 (0.05) | 1.48 (0.06) | 1.6 (0.05) | 1.68 (0.05) | 1.71 (0.05) | 1.76 (0.05) | 1.79 (0.05) | 1.69 (0.05) | 1.96 (0.05) | 1.78 (0.04) | 1.87 (0.04) | 1.79 (0.04) | 1.83 (0.04) | 1.87 (0.04) | 1.96 (0.04) | 1.98 (0.04) | 1.77 (0.04) | 2.02 (0.04) | 1.97 (0.04) | 2.01 (0.04) | 2.18 (0.04) | 1.98 (0.04) |
|  | South | 1.96 (0.1) | 2.4 (0.1) | 2.23 (0.1) | 2.6 (0.1) | 2.45 (0.08) | 2.29 (0.09) | 2.3 (0.09) | 2.52 (0.09) | 2.68 (0.09) | 2.65 (0.09) | 2.97 (0.09) | 2.33 (0.08) | 2.35 (0.07) | 2.51 (0.07) | 2.44 (0.07) | 2.6 (0.07) | 2.91 (0.07) | 2.78 (0.07) | 2.73 (0.07) | 2.7 (0.07) | 2.96 (0.06) | 2.8 (0.07) | 2.73 (0.06) | 2.86 (0.07) |
|  |  |  |  |  |  |  |  |  |  |  |  |  |  |  |  |  |  |  |  |  |  |  |  |  |  |
| **Prostate cancer** | **Brazil** | 10.96 (0.14) | 11.83 (0.15) | 12.58 (0.15) | 12.57 (0.15) | 11.39 (0.13) | 12.03 (0.14) | 12.4 (0.14) | 13.13 (0.14) | 13.84 (0.14) | 14.39 (0.14) | 15.29 (0.15) | 13.34 (0.13) | 13.57 (0.13) | 13.26 (0.12) | 13.25 (0.12) | 13.5 (0.12) | 13.65 (0.12) | 13.73 (0.12) | 13.59 (0.12) | 13.38 (0.11) | 13.25 (0.11) | 13.12 (0.11) | 12.73 (0.1) | 12.53 (0.1) |
|  | North | 6.46 (0.48) | 7.34 (0.51) | 6.52 (0.47) | 7.23 (0.49) | 7.33 (0.46) | 7.11 (0.45) | 7.42 (0.45) | 9.11 (0.5) | 8.92 (0.49) | 8.95 (0.48) | 10.72 (0.52) | 9.47 (0.45) | 11.04 (0.48) | 10.29 (0.46) | 10.14 (0.43) | 10.71 (0.44) | 12.26 (0.47) | 13.79 (0.51) | 14.68 (0.52) | 15.07 (0.51) | 13.33 (0.47) | 14.6 (0.48) | 13.56 (0.46) | 13.13 (0.44) |
|  | Northeast | 5.93 (0.19) | 6.74 (0.2) | 7.24 (0.21) | 7.41 (0.21) | 7.21 (0.2) | 7.16 (0.19) | 8.29 (0.21) | 8.65 (0.21) | 9.58 (0.22) | 10.95 (0.23) | 13.55 (0.26) | 13.12 (0.24) | 13.6 (0.24) | 13.32 (0.24) | 12.51 (0.22) | 13.16 (0.22) | 12.99 (0.22) | 15.01 (0.25) | 14.99 (0.24) | 15.39 (0.24) | 15.24 (0.24) | 15.43 (0.24) | 14.92 (0.23) | 14.83 (0.22) |
|  | Center-West | 11.63 (0.64) | 13.8 (0.69) | 13.68 (0.68) | 16.08 (0.73) | 13.02 (0.6) | 14.09 (0.62) | 13.63 (0.6) | 15.16 (0.63) | 15.33 (0.63) | 16.01 (0.64) | 16.86 (0.65) | 13.49 (0.52) | 14.18 (0.52) | 13.58 (0.49) | 14.53 (0.49) | 15.15 (0.5) | 14.84 (0.5) | 15.15 (0.49) | 15.91 (0.49) | 14.51 (0.46) | 14.85 (0.45) | 14.13 (0.43) | 13.36 (0.41) | 13.28 (0.4) |
|  | Southeast | 13.79 (0.24) | 14.75 (0.25) | 15.71 (0.25) | 15.14 (0.25) | 13.33 (0.22) | 14.19 (0.22) | 14.52 (0.22) | 15.24 (0.23) | 15.86 (0.23) | 16.04 (0.23) | 15.65 (0.22) | 13.26 (0.19) | 13.4 (0.18) | 13.0 (0.18) | 13.07 (0.18) | 13.09 (0.18) | 13.51 (0.18) | 12.47 (0.16) | 12.12 (0.16) | 11.97 (0.16) | 12.1 (0.15) | 11.78 (0.15) | 11.45 (0.14) | 11.32 (0.14) |
|  | South | 14.81 (0.42) | 15.2 (0.43) | 16.78 (0.44) | 16.75 (0.44) | 15.28 (0.39) | 16.73 (0.41) | 16.13 (0.4) | 16.83 (0.41) | 18.11 (0.42) | 18.18 (0.42) | 19.01 (0.43) | 15.25 (0.34) | 14.6 (0.33) | 14.66 (0.32) | 15.81 (0.33) | 15.82 (0.33) | 15.52 (0.33) | 14.86 (0.3) | 14.49 (0.29) | 13.48 (0.28) | 13.02 (0.26) | 12.84 (0.26) | 12.77 (0.25) | 12.29 (0.24) |
|  |  |  |  |  |  |  |  |  |  |  |  |  |  |  |  |  |  |  |  |  |  |  |  |  |  |
| **Penile cancer** | **Brazil** | 0.28 (0.02) | 0.33 (0.02) | 0.32 (0.02) | 0.34 (0.02) | 0.3 (0.02) | 0.3 (0.02) | 0.31 (0.02) | 0.33 (0.02) | 0.37 (0.02) | 0.33 (0.02) | 0.37 (0.02) | 0.35 (0.02) | 0.38 (0.02) | 0.34 (0.02) | 0.38 (0.02) | 0.35 (0.02) | 0.39 (0.02) | 0.39 (0.02) | 0.37 (0.02) | 0.38 (0.02) | 0.37 (0.02) | 0.39 (0.02) | 0.39 (0.02) | 0.38 (0.02) |
|  | North | 0.12 (0.06) | 0.59 (0.14) | 0.16 (0.06) | 0.22 (0.08) | 0.17 (0.07) | 0.38 (0.1) | 0.3 (0.08) | 0.38 (0.1) | 0.34 (0.09) | 0.31 (0.08) | 0.35 (0.09) | 0.42 (0.09) | 0.6 (0.11) | 0.35 (0.08) | 0.37 (0.08) | 0.54 (0.1) | 0.6 (0.1) | 0.62 (0.1) | 0.68 (0.11) | 0.66 (0.1) | 0.74 (0.11) | 0.77 (0.11) | 0.58 (0.09) | 0.55 (0.09) |
|  | Northeast | 0.21 (0.04) | 0.23 (0.04) | 0.27 (0.04) | 0.35 (0.05) | 0.26 (0.04) | 0.31 (0.04) | 0.31 (0.04) | 0.29 (0.04) | 0.43 (0.05) | 0.39 (0.04) | 0.48 (0.05) | 0.42 (0.04) | 0.58 (0.05) | 0.45 (0.05) | 0.54 (0.05) | 0.47 (0.04) | 0.53 (0.05) | 0.53 (0.05) | 0.53 (0.05) | 0.47 (0.04) | 0.51 (0.04) | 0.55 (0.04) | 0.56 (0.04) | 0.56 (0.04) |
|  | Center-West | 0.53 (0.13) | 0.41 (0.11) | 0.59 (0.13) | 0.57 (0.13) | 0.41 (0.1) | 0.33 (0.09) | 0.37 (0.1) | 0.22 (0.07) | 0.35 (0.09) | 0.66 (0.12) | 0.56 (0.11) | 0.42 (0.09) | 0.38 (0.08) | 0.33 (0.08) | 0.51 (0.09) | 0.41 (0.08) | 0.51 (0.09) | 0.34 (0.07) | 0.46 (0.08) | 0.55 (0.09) | 0.36 (0.07) | 0.46 (0.08) | 0.5 (0.08) | 0.36 (0.06) |
|  | Southeast | 0.32 (0.03) | 0.34 (0.04) | 0.33 (0.04) | 0.3 (0.03) | 0.32 (0.03) | 0.3 (0.03) | 0.32 (0.03) | 0.37 (0.03) | 0.35 (0.03) | 0.29 (0.03) | 0.33 (0.03) | 0.28 (0.03) | 0.26 (0.03) | 0.3 (0.03) | 0.28 (0.03) | 0.27 (0.03) | 0.3 (0.03) | 0.32 (0.03) | 0.26 (0.02) | 0.29 (0.02) | 0.27 (0.02) | 0.27 (0.02) | 0.28 (0.02) | 0.28 (0.02) |
|  | South | 0.26 (0.05) | 0.35 (0.06) | 0.32 (0.06) | 0.42 (0.07) | 0.33 (0.06) | 0.26 (0.05) | 0.29 (0.05) | 0.31 (0.05) | 0.38 (0.06) | 0.26 (0.05) | 0.25 (0.05) | 0.37 (0.05) | 0.3 (0.05) | 0.29 (0.05) | 0.36 (0.05) | 0.24 (0.04) | 0.25 (0.04) | 0.3 (0.04) | 0.3 (0.04) | 0.32 (0.04) | 0.3 (0.04) | 0.3 (0.04) | 0.3 (0.04) | 0.32 (0.04) |
|  |  |  |  |  |  |  |  |  |  |  |  |  |  |  |  |  |  |  |  |  |  |  |  |  |  |
| **Testis cancer** | **Brazil** | 0.24 (0.02) | 0.22 (0.02) | 0.26 (0.02) | 0.2 (0.02) | 0.21 (0.02) | 0.21 (0.02) | 0.23 (0.02) | 0.27 (0.02) | 0.25 (0.02) | 0.27 (0.02) | 0.28 (0.02) | 0.26 (0.02) | 0.28 (0.02) | 0.26 (0.02) | 0.27 (0.02) | 0.28 (0.02) | 0.29 (0.02) | 0.31 (0.02) | 0.29 (0.02) | 0.32 (0.02) | 0.27 (0.02) | 0.35 (0.02) | 0.33 (0.02) | 0.38 (0.02) |
|  | North | 0.15 (0.07) | 0.09 (0.05) | 0.15 (0.06) | 0.13 (0.05) | 0.12 (0.04) | 0.18 (0.06) | 0.14 (0.05) | 0.29 (0.08) | 0.13 (0.05) | 0.28 (0.08) | 0.13 (0.05) | 0.21 (0.06) | 0.21 (0.06) | 0.31 (0.06) | 0.23 (0.05) | 0.34 (0.06) | 0.39 (0.07) | 0.24 (0.05) | 0.17 (0.04) | 0.18 (0.05) | 0.21 (0.05) | 0.34 (0.06) | 0.35 (0.06) | 0.27 (0.05) |
|  | Northeast | 0.12 (0.03) | 0.06 (0.02) | 0.08 (0.02) | 0.07 (0.02) | 0.08 (0.02) | 0.07 (0.02) | 0.09 (0.02) | 0.12 (0.02) | 0.1 (0.02) | 0.12 (0.02) | 0.19 (0.03) | 0.12 (0.02) | 0.16 (0.03) | 0.13 (0.02) | 0.14 (0.02) | 0.14 (0.02) | 0.17 (0.03) | 0.17 (0.02) | 0.15 (0.02) | 0.17 (0.02) | 0.1 (0.02) | 0.23 (0.03) | 0.16 (0.02) | 0.22 (0.03) |
|  | Center-West | 0.1 (0.05) | 0.26 (0.08) | 0.19 (0.07) | 0.18 (0.06) | 0.21 (0.07) | 0.18 (0.06) | 0.14 (0.05) | 0.2 (0.07) | 0.32 (0.08) | 0.33 (0.08) | 0.23 (0.07) | 0.3 (0.07) | 0.24 (0.06) | 0.18 (0.05) | 0.23 (0.05) | 0.23 (0.05) | 0.12 (0.04) | 0.23 (0.05) | 0.25 (0.06) | 0.35 (0.07) | 0.23 (0.05) | 0.23 (0.05) | 0.29 (0.06) | 0.32 (0.06) |
|  | Southeast | 0.26 (0.03) | 0.24 (0.03) | 0.28 (0.03) | 0.23 (0.03) | 0.22 (0.02) | 0.23 (0.02) | 0.27 (0.03) | 0.26 (0.03) | 0.25 (0.03) | 0.26 (0.03) | 0.3 (0.03) | 0.28 (0.03) | 0.31 (0.03) | 0.25 (0.02) | 0.3 (0.03) | 0.28 (0.02) | 0.26 (0.02) | 0.32 (0.03) | 0.35 (0.03) | 0.33 (0.03) | 0.32 (0.03) | 0.38 (0.03) | 0.34 (0.03) | 0.43 (0.03) |
|  | South | 0.49 (0.07) | 0.47 (0.06) | 0.57 (0.07) | 0.34 (0.05) | 0.45 (0.06) | 0.46 (0.06) | 0.47 (0.06) | 0.6 (0.07) | 0.51 (0.06) | 0.55 (0.07) | 0.5 (0.06) | 0.44 (0.05) | 0.46 (0.06) | 0.53 (0.06) | 0.49 (0.06) | 0.54 (0.06) | 0.61 (0.06) | 0.64 (0.06) | 0.51 (0.06) | 0.62 (0.06) | 0.53 (0.06) | 0.59 (0.06) | 0.69 (0.06) | 0.62 (0.06) |

**Online Resource 3** Distribution of annual age-standardized mortality rates for urological cancers in Brazilian men from 1996 to 2019
