# Supplementary material for: Epidemiology of Urological Cancers in Brazil: Trends in Mortality Rates Over More Than Two Decades
Source: J Epidemiol Glob Health. 2022 May 31;12(3):239–47. doi: 10.1007/s44197-022-00042-8 (PMC9470798; doi:10.1007/s44197-022-00042-8)
Supplement: Supplementary file 4 — Online Resource 4: Distribution of the annual age-standardized mortality rates for bladder and kidney cancers in Brazilian women from 1996 to 2019 (DOCX 19 KB) [file 44197_2022_42_MOESM4_ESM.docx]

|  | **Region** | **1996** | **1997** | **1998** | **1999** | **2000** | **2001** | **2002** | **2003** | **2004** | **2005** | **2006** | **2007** | **2008** | **2009** | **2010** | **2011** | **2012** | **2013** | **2014** | **2015** | **2016** | **2017** | **2018** | **2019** |
| --- | --- | --- | --- | --- | --- | --- | --- | --- | --- | --- | --- | --- | --- | --- | --- | --- | --- | --- | --- | --- | --- | --- | --- | --- | --- |
| **Bladder cancer** |  |  |  |  |  |  |  |  |  |  |  |  |  |  |  |  |  |  |  |  |  |  |  |  |  |
| Female | **Brazil** | 0.71 (0.03) | 0.78 (0.03) | 0.76 (0.03) | 0.82 (0.03) | 0.71 (0.03) | 0.76 (0.03) | 0.84 (0.03) | 0.81 (0.03) | 0.85 (0.03) | 0.9 (0.03) | 0.85 (0.03) | 0.78 (0.03) | 0.78 (0.03) | 0.78 (0.03) | 0.76 (0.03) | 0.81 (0.03) | 0.85 (0.03) | 0.83 (0.03) | 0.9 (0.03) | 0.87 (0.03) | 0.84 (0.02) | 0.87 (0.02) | 0.88 (0.02) | 0.84 (0.02) |
|  | North | 0.35 (0.11) | 0.18 (0.08) | 0.29 (0.1) | 0.32 (0.11) | 0.49 (0.12) | 0.4 (0.11) | 0.21 (0.07) | 0.42 (0.1) | 0.41 (0.1) | 0.44 (0.11) | 0.53 (0.11) | 0.38 (0.09) | 0.52 (0.11) | 0.29 (0.07) | 0.36 (0.08) | 0.52 (0.1) | 0.44 (0.09) | 0.64 (0.11) | 0.59 (0.1) | 0.72 (0.11) | 0.59 (0.1) | 0.56 (0.09) | 0.76 (0.1) | 0.51 (0.08) |
|  | Northeast | 0.31 (0.04) | 0.33 (0.04) | 0.36 (0.04) | 0.49 (0.05) | 0.46 (0.05) | 0.41 (0.04) | 0.52 (0.05) | 0.42 (0.04) | 0.46 (0.05) | 0.52 (0.05) | 0.5 (0.05) | 0.53 (0.05) | 0.46 (0.04) | 0.59 (0.05) | 0.56 (0.04) | 0.53 (0.04) | 0.53 (0.04) | 0.61 (0.04) | 0.68 (0.05) | 0.67 (0.05) | 0.65 (0.04) | 0.65 (0.04) | 0.65 (0.04) | 0.6 (0.04) |
|  | Center-West | 0.71 (0.15) | 0.77 (0.16) | 0.77 (0.15) | 0.77 (0.16) | 0.77 (0.14) | 1.03 (0.16) | 0.88 (0.15) | 0.88 (0.14) | 0.77 (0.13) | 1.25 (0.17) | 0.96 (0.15) | 0.92 (0.13) | 1.03 (0.13) | 0.85 (0.12) | 0.82 (0.11) | 0.87 (0.11) | 1.07 (0.13) | 0.76 (0.1) | 0.97 (0.11) | 0.91 (0.11) | 0.94 (0.1) | 0.79 (0.09) | 0.97 (0.1) | 0.81 (0.09) |
|  | Southeast | 0.87 (0.05) | 1.0 (0.06) | 0.95 (0.05) | 1.01 (0.06) | 0.86 (0.05) | 0.9 (0.05) | 1.02 (0.05) | 0.98 (0.05) | 1.07 (0.05) | 1.06 (0.05) | 1.0 (0.05) | 0.87 (0.04) | 0.91 (0.04) | 0.89 (0.04) | 0.86 (0.04) | 0.92 (0.04) | 0.98 (0.04) | 0.91 (0.04) | 1.01 (0.04) | 0.92 (0.04) | 0.91 (0.04) | 0.97 (0.04) | 0.96 (0.04) | 0.94 (0.04) |
|  | South | 1.03 (0.1) | 1.08 (0.1) | 1.08 (0.1) | 1.0 (0.1) | 0.81 (0.08) | 0.99 (0.09) | 1.04 (0.09) | 1.05 (0.09) | 1.02 (0.09) | 1.14 (0.09) | 1.1 (0.09) | 1.0 (0.08) | 0.9 (0.07) | 0.89 (0.07) | 0.86 (0.07) | 1.05 (0.07) | 1.03 (0.07) | 1.03 (0.07) | 1.01 (0.07) | 1.07 (0.07) | 0.94 (0.06) | 1.04 (0.07) | 1.01 (0.06) | 1.05 (0.07) |
| **Kidney cancer** |  |  |  |  |  |  |  |  |  |  |  |  |  |  |  |  |  |  |  |  |  |  |  |  |  |
| Female | **Brazil** | 0.76 (0.03) | 0.73 (0.03) | 0.82 (0.03) | 0.72 (0.03) | 0.81 (0.03) | 0.76 (0.03) | 0.87 (0.03) | 0.82 (0.03) | 0.82 (0.03) | 0.82 (0.03) | 0.89 (0.03) | 0.77 (0.03) | 0.81 (0.03) | 0.87 (0.03) | 0.84 (0.03) | 0.81 (0.03) | 0.91 (0.03) | 0.88 (0.03) | 0.87 (0.03) | 0.95 (0.03) | 0.93 (0.02) | 0.95 (0.02) | 0.95 (0.02) | 0.94 (0.02) |
|  | North | 0.3 (0.11) | 0.52 (0.08) | 0.67 (0.1) | 0.59 (0.11) | 0.52 (0.12) | 0.42 (0.11) | 0.43 (0.07) | 0.47 (0.1) | 0.52 (0.1) | 0.42 (0.11) | 0.59 (0.11) | 0.51 (0.09) | 0.59 (0.11) | 0.75 (0.07) | 0.46 (0.08) | 0.43 (0.1) | 0.89 (0.09) | 0.77 (0.11) | 0.85 (0.1) | 0.7 (0.11) | 0.82 (0.1) | 0.82 (0.09) | 1.0 (0.1) | 0.88 (0.08) |
|  | Northeast | 0.36 (0.04) | 0.28 (0.04) | 0.42 (0.04) | 0.4 (0.05) | 0.36 (0.05) | 0.39 (0.04) | 0.42 (0.05) | 0.5 (0.04) | 0.48 (0.05) | 0.52 (0.05) | 0.63 (0.05) | 0.52 (0.05) | 0.58 (0.04) | 0.71 (0.05) | 0.67 (0.04) | 0.56 (0.04) | 0.61 (0.04) | 0.68 (0.04) | 0.59 (0.05) | 0.75 (0.05) | 0.62 (0.04) | 0.77 (0.04) | 0.71 (0.04) | 0.67 (0.04) |
|  | Center-West | 0.43 (0.15) | 0.57 (0.16) | 0.62 (0.15) | 0.76 (0.16) | 0.68 (0.14) | 0.71 (0.16) | 1.04 (0.15) | 0.99 (0.14) | 1.02 (0.13) | 0.95 (0.17) | 1.2 (0.15) | 1.07 (0.13) | 0.64 (0.13) | 0.87 (0.12) | 0.7 (0.11) | 0.89 (0.11) | 1.01 (0.13) | 0.74 (0.1) | 1.12 (0.11) | 1.05 (0.11) | 0.94 (0.1) | 0.94 (0.09) | 0.9 (0.1) | 0.97 (0.09) |
|  | Southeast | 0.87 (0.05) | 0.86 (0.06) | 0.88 (0.05) | 0.73 (0.06) | 0.92 (0.05) | 0.82 (0.05) | 0.98 (0.05) | 0.85 (0.05) | 0.87 (0.05) | 0.82 (0.05) | 0.89 (0.05) | 0.79 (0.04) | 0.84 (0.04) | 0.85 (0.04) | 0.83 (0.04) | 0.81 (0.04) | 0.95 (0.04) | 0.88 (0.04) | 0.88 (0.04) | 0.93 (0.04) | 0.97 (0.04) | 0.95 (0.04) | 0.96 (0.04) | 0.95 (0.04) |
|  | South | 1.36 (0.1) | 1.21 (0.1) | 1.39 (0.1) | 1.28 (0.1) | 1.36 (0.08) | 1.31 (0.09) | 1.37 (0.09) | 1.32 (0.09) | 1.25 (0.09) | 1.42 (0.09) | 1.33 (0.09) | 1.08 (0.08) | 1.21 (0.07) | 1.21 (0.07) | 1.28 (0.07) | 1.29 (0.07) | 1.28 (0.07) | 1.29 (0.07) | 1.18 (0.07) | 1.27 (0.07) | 1.32 (0.06) | 1.29 (0.07) | 1.33 (0.06) | 1.32 (0.07) |

**Online Resource 4** Distribution of the annual age-standardized mortality rates for bladder and kidney cancers in Brazilian women from 1996 to 2019
